# Selective Vapor Condensation for the Synthesis and Assembly of Spherical Colloids with a Precise Rough Patch

**DOI:** 10.1021/jacsau.3c00812

**Published:** 2024-03-04

**Authors:** Kennedy A. Guillot, Philip J. Brahana, Ahmed Al Harraq, Nduka D. Ogbonna, Nicholas S. Lombardo, Jimmy Lawrence, Yaxin An, Michael G. Benton, Bhuvnesh Bharti

**Affiliations:** Cain Department of Chemical Engineering, Louisiana State University, Baton Rouge, Louisiana 70803, United States

**Keywords:** colloidal assembly, patchy particles, directed assembly, vapor−liquid equilibria, condensation, surface interactions, molecular dynamics

## Abstract

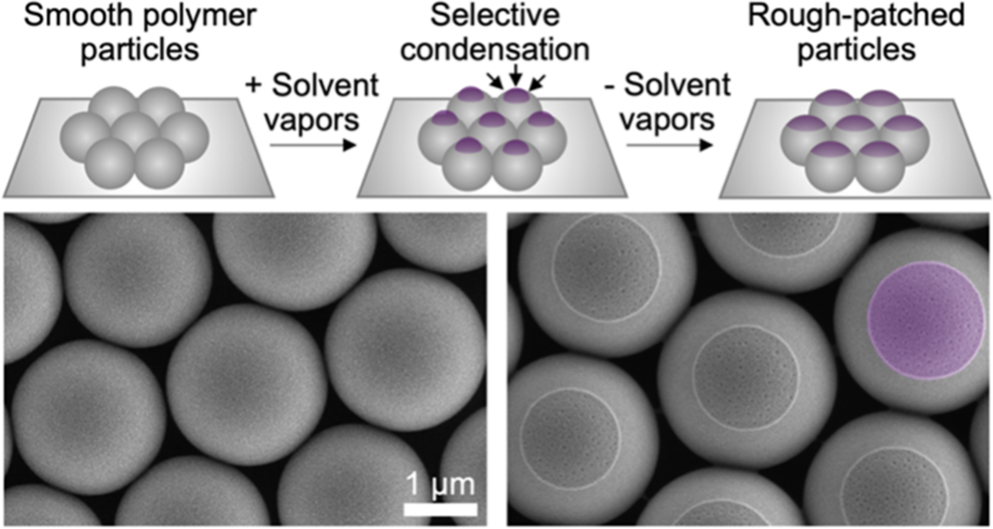

Patchy particles occupy an increasingly important space in soft matter research due to their ability to assemble into intricate phases and states. Being able to fine-tune the interactions among these particles is essential to understanding the principles governing the self-assembly processes. However, current fabrication techniques often yield patches that deviate chemically and physically from the native particles, impeding the identification of the driving forces behind self-assembly. To overcome this challenge, we propose a new approach to synthesizing spherical colloids with a well-defined rough patch on their surface. By treating polystyrene microspheres with vapors of a good solvent, here an acetone–water mixture, we achieve selective polymer corrugation on the particle surface resulting in a chemically similar yet rough surface patch. The key step is the selective condensation of the acetone–water vapors on the apex of the polystyrene microparticles immobilized on a substrate, which leads to rough patch formation. We leverage the ability to tune the vapor–liquid equilibrium of the volatile acetone–water mixture to precisely control the polymer corrugation on the particle surface. We demonstrate the dependence of patch formation on particle and substrate wettability, with the condensation occurring on the particle apex only when it is more wettable than the substrate, which is consistent with Volmer’s classical nucleation theory. By combining experiments and molecular dynamics simulations, we identify the role of the rough patch in the depletion interaction-driven self-assembly of the microspheres, which is crucial for designing programmable supracolloidal structures.

Patchy colloids are micro- and nanoparticles characterized by spatially defined and distinct surface inhomogeneities called “patches”.^1−5^ These patches introduce anisotropy in orientation-dependent interparticle interactions, enabling precise control over assembly and propulsion behaviors.^6−12^ A notable example of a patchy colloid is the spherical Janus particle, where the hemispherical patch possesses physicochemical properties that differ from the core particle.^13^ Possessing dynamical characteristics similar to atoms, these anisotropic colloids function as pioneering building blocks in numerous soft matter research areas, acting as a versatile synthetic platform for investigating self-assembly mechanisms at atomic and molecular scales.^14−22^ Thus, designing colloids with well-defined patch-to-patch interactions is fundamental to programming the self-assembly of patchy colloids into supracolloidal domains.

Assembly of colloidal particles can be directed into ordered domains by precise control over the operational interparticle interactions, which are strongly dependent on the local physicochemical characteristics of the particles, such as surface roughness. Previously, electrostatic interactions, capillary attraction, and external electromagnetic fields have been used to program the assembly of patchy colloids in bulk as well as at interfaces.^1,15,23−31^ One of the most versatile methods to program colloidal phase behavior is via depletion interactions.^32,33^ The depletion attraction is triggered by the presence of nonadsorbing polymer or nanoparticles surrounding the larger colloidal particles.^34^ Thus, a pair of interacting particles share an excluded volume which is “depleted” of the nonadsorbing objects due to entropic considerations. One of the major factors influencing the depletion attraction is the surface roughness of the interacting particles. It has been shown that the depletion attraction is suppressed upon increasing the surface roughness, and completely vanishes when roughness approaches the depletant size.^33,35−37^ The difference in the degree of attraction between smooth and rough surfaces has been used to assemble micellar structures of dumbbell-shaped colloids composed of one rough and one smooth lobe.^33,38^ While previous studies have established surface roughness as one of the key factors influencing the assembly process, the dumbbell shape of the particles hindered the complete delineation of the contributions of the particle shape from the surface roughness on the assembly process. No model system of spherical particles with a discrete rough surface patch is currently available, primarily due to a lack of control over the surface roughness of the particles synthesized either with top-down soft-lithography or bottom-up bulk synthesis.^39,40^ Current approaches of introducing surface roughness are primarily limited to the binding of smaller-sized particles onto a larger core.^36,41^ Such approaches do not provide control over the binding location of the small particle and lead to the formation of isotopically rough colloidal particles. Hence, a new methodology for the fabrication of spherical colloids with spatially defined rough patches is highly desirable.

In this paper, we introduce preferential solvent vapor nucleation on a substrate as a method to precisely engrave a rough patch onto a spherical polymeric microparticle. We introduce the preferential condensation of a good solvent on the apex of a smooth polymeric microparticle immobilized on the substrate and the corresponding selective structural reconfiguration and partial dissolution of the polymeric chains at the particle apex as robust methods for large-scale microfabrication of colloidal particles with discrete rough patches. Here, the patch on the particle surface is distinct from the core only in physical roughness while maintaining the spherical shape and chemical properties of the original particle.

Polymer dissolution, a phenomenon driven by solvent diffusion and polymer chain disentanglement, induces full or partial miscibility of the polymer in a suitable solvent.^42−44^ Interaction with solvent molecules causes the polymer chains to swell, and a subsequent evaporation of the solvent typically leaves behind a roughened surface. Harnessing these fundamental insights, we have developed a versatile technique to generate uniform and spatially defined rough patches on the surfaces of non-cross-linked, smooth polystyrene (PS) microspheres. We use an acetone–water mixture as a solvent with tunable vapor–liquid equilibrium (VLE) characteristics and program its condensation behavior to synthesize the spherical patchy particles. The key mechanistic step is the selective nucleation and condensation of vapors of the acetone/water mixture on the apex of the PS sphere immobilized on a substrate to form a rough patch. We first demonstrate the capability of our VLE-based polymer corrugation method to synthesize PS microspheres with discrete rough patches and discuss the corresponding mechanism. Second, we investigate the impact of the presence of the rough patches on the depletion attraction-induced self-assembly of the spherical patchy particles. We combine experiments with molecular dynamics simulations to uncover the role of rough patches in directing the self-assembly of colloids via depletion attraction.

## Results and Discussion

**Scheme 1:**
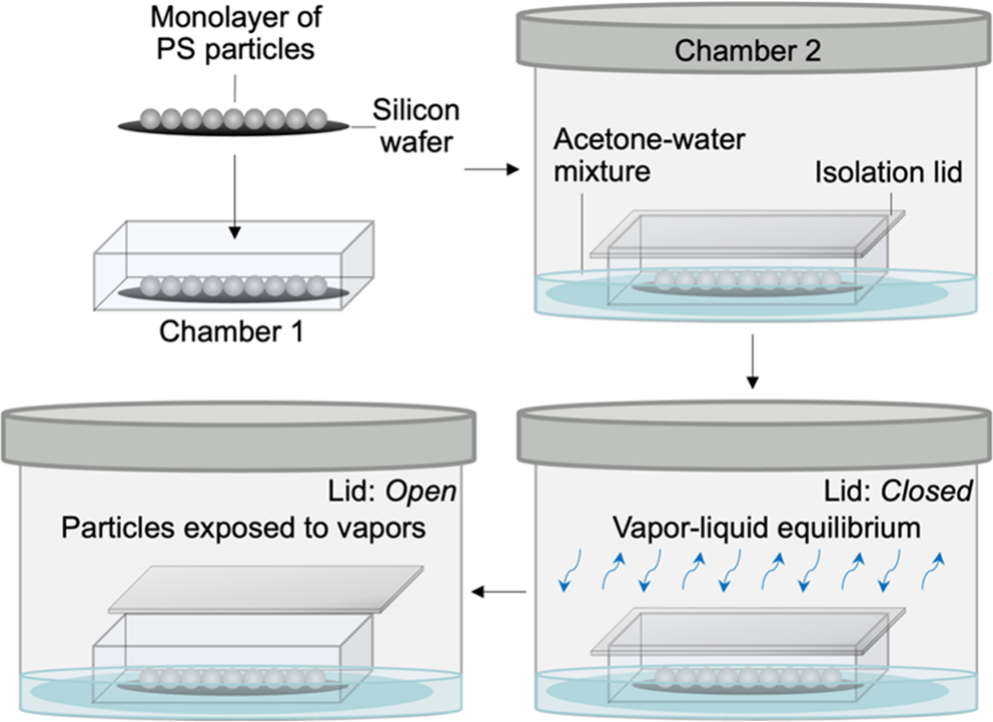
Schematic Representation of the Experimental Procedure Used for the Synthesis of Spherical Microparticles with Discrete Rough Patches A silicon wafer containing a monolayer of closed-packed PS microspheres is sealed in Chamber 1 using an airtight isolation lid. Chamber 1 is then placed within a larger Chamber 2 that is filled with acetone-water mixture of known liquid-phase composition. After attaining VLE within Chamber 2, the inner Chamber 1 is opened using a remote mechanism, and the microparticles are exposed to the vapors of acetone-water mixture, driving the preferential condensation of the vapors on the apex of the particles. The composition of the liquid droplet condensed onto the particle is identical to the bulk liquid acetone–vapor mixture due to the dynamic nature of the established VLE.

We use PS microspheres as model particles and acetone–water mixtures as the corresponding good solvent. The non-crosslinked PS microparticles with a radius (σ) of ∼1.1 μm (Figure S1) were synthesized using dispersion polymerization.^45−47^ A monolayer of these particles was deposited onto a silicon wafer by using a Langmuir–Blodgett trough. To introduce rough patches on the particle surfaces, a custom-built experimental setup was used, consisting of two chambers as shown in Scheme 1. The silicon wafer coated with the monolayer of PS particles was placed in Chamber 1 of volume ∼115 cm^3^ and sealed using an airtight isolation lid. Chamber 1 was then placed within a larger Chamber 2 of volume ∼1550 cm^3^. Chamber 2 was then filled with 100 mL of acetone–water liquid mixture of known molar composition, sealed, and allowed to equilibrate for 1 h to attain VLE at room temperature (∼20 °C). After equilibration, the inner chamber was opened using a remote mechanism, exposing the PS particles to the vapors of the acetone–water mixture (Figure S2). The equilibration time before opening Chamber 1 was necessary to achieve VLE (Figure S3), which is critical for the subsequent nucleation and condensation of vapors on the particle apex. The exposed particles were taken out after opening the two chambers, during which the acetone–water droplets condensed on the particles evaporate, leaving behind well-defined rough patches (discussed later).

### Physicochemical Characteristics of the Patchy Particles

**Figure 1:**
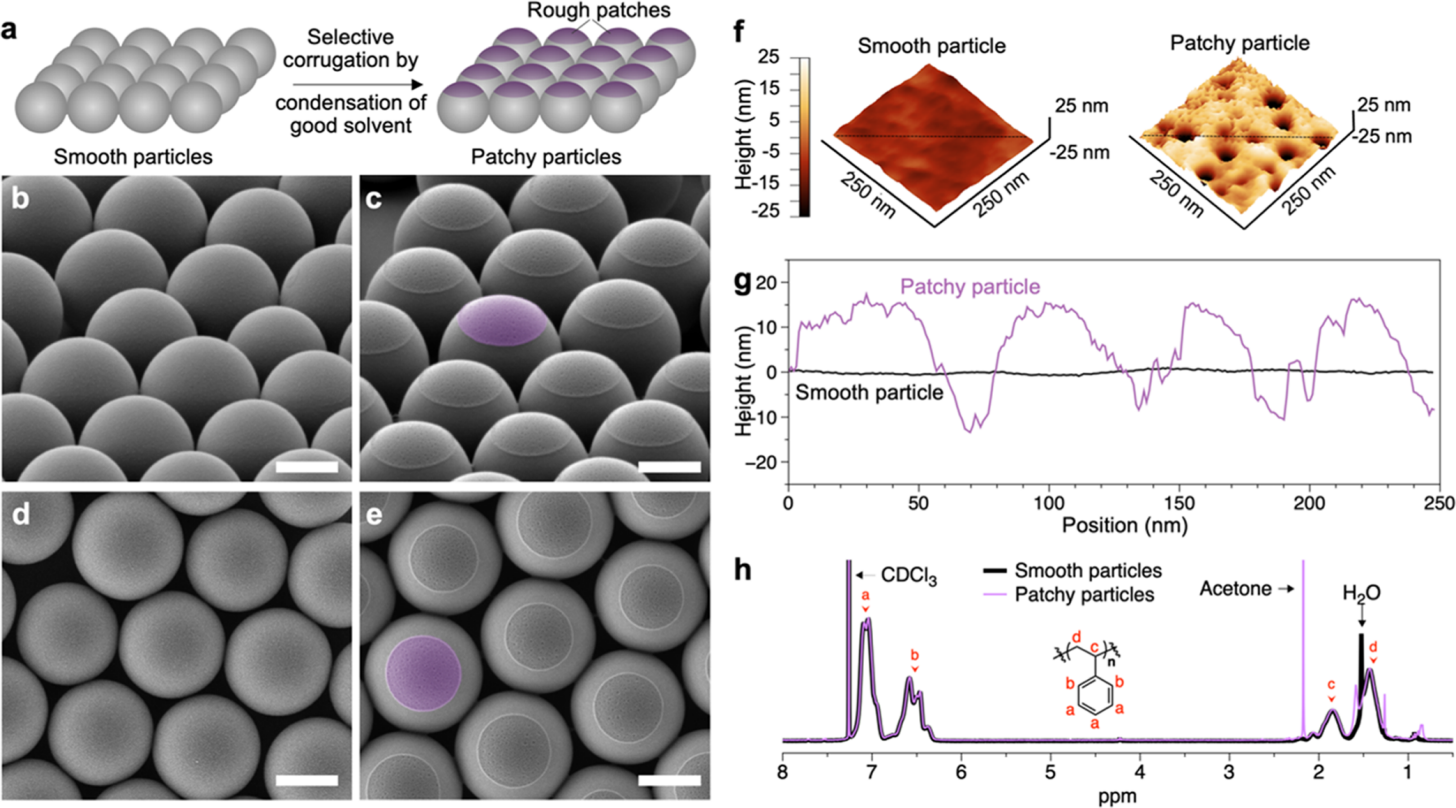
(a) Schematic showing the selective condensation of acetone–water vapor mixture on the apex of the smooth particles and the corresponding formation of rough patches. (b-e) Perspective and top view of the PS particles using SEM. (b, d) Particles before and (c, e) after 30 min of exposure to acetone–water vapors with ϕ_L_ = 0.36. The SEM images in (c) and (e) show the formation of rough patches on PS particles upon exposure to vapors of acetone–water mixture. Here, one patch is false colored (purple) for clarity purpose and scale bars in (b–e) are 1 μm. (f) 3D reconstructed AFM images and (g) the corresponding height profiles of smooth PS particle and the rough patch formed after exposure to acetone–water vapors. The height profiles are obtained from the horizontal lines shown in the 3D AFM images in (f). (h) ^1^H NMR spectrum of the particles before and after exposure to the vapors of the acetone–water mixture, showing near-identical chemical composition in the two cases. The rough particles showed the presence of acetone, which likely remained trapped in the particles after the patch formation process.

The vapors condense onto the apex of the smooth PS particles and lead to selective corrugation of the polymer and formation of rough patches (Figure 1a). We use scanning electron microscopy (SEM) to identify transformations occurring in the PS particles after exposure to the acetone–water vapors as a function of vapor exposure duration, *t* (Figure 1b–e). The composition of the acetone–water mixture is expressed as the relative mole fraction of acetone in the liquid phase (ϕ_L_). Note that the composition of the droplet which condenses onto the apex of the PS particle was identical to the composition of the liquid phase, due to established VLE.^48^ After *t* = 30 min of exposure to acetone–water vapors (ϕ_L_ = 0.36), every PS particle in the monolayer shows the formation of a rough patch (Figure 1c,e). All observed patches were formed on the apex of the particles and show a high degree of circularity, which can be attributed to the surface tension of the acetone–water mixture droplet condensed on the particle apex (γ_lv_ ∼ 30 mN m^–1^).

We quantify the degree of roughness of the patch formed on the surface of PS particles using atomic force microscopy (AFM) as shown in Figure 1f–g (see the Methods section for experimental details). The 3D reconstructed AFM images show a smooth surface of the isotropic particles with <1 nm of variation in the height profile (Figure 1g). In contrast, the 3D profile of the patch formed on the PS particle shows a high degree of roughness, with variation in height up to 25 nm (Figure 1g). The surface roughness on the patch is due to the physical transformation of the polystyrene, not a chemical one, triggered by lateral instability. This instability arises from competing entropic and energetic forces. Entropy, in this context, refers to the preference of physically entangled polymer chains for expansion. Energetics, on the other hand, refers to the enthalpy change during polymer swelling in the acetone–water mixture. As the polymer chain swells, the impact of higher molar mass species within the distribution of 8 to 200 kDa (Figure S4) becomes more pronounced due to chain–chain entanglement. Note that the entanglement molecular weight of the polystyrene is ∼12 kDa, and the radius of gyration at ∼150 kDa is ∼12 nm. This entanglement-induced instability manifests as surface roughening on a scale of tens of nanometers, a feature size consistent with our AFM height profile of the patch (Figure 1g). Importantly, size exclusion chromatography, proton nuclear magnetic resonance (^1^H NMR), and attenuated total reflection – Fourier transform infrared (ATR-FTIR) spectroscopy analyses of both pristine and patchy particles confirm that the molar mass distribution and the chemical composition of the PS particles remain identical before and after exposure to the acetone–water vapors (Figures 1h and S4). The ^1^H NMR analysis indicated the presence of residual water in the smooth particles and acetone as well as water in the rough particles, as expected. Overall, these findings underscore our conclusion that the observed surface roughness arises from physical changes during the swelling process (Figure S5) rather than from the loss of a lower-molecular-weight species or surface oxidation.

### Mechanism of Patch Formation

**Figure 2:**
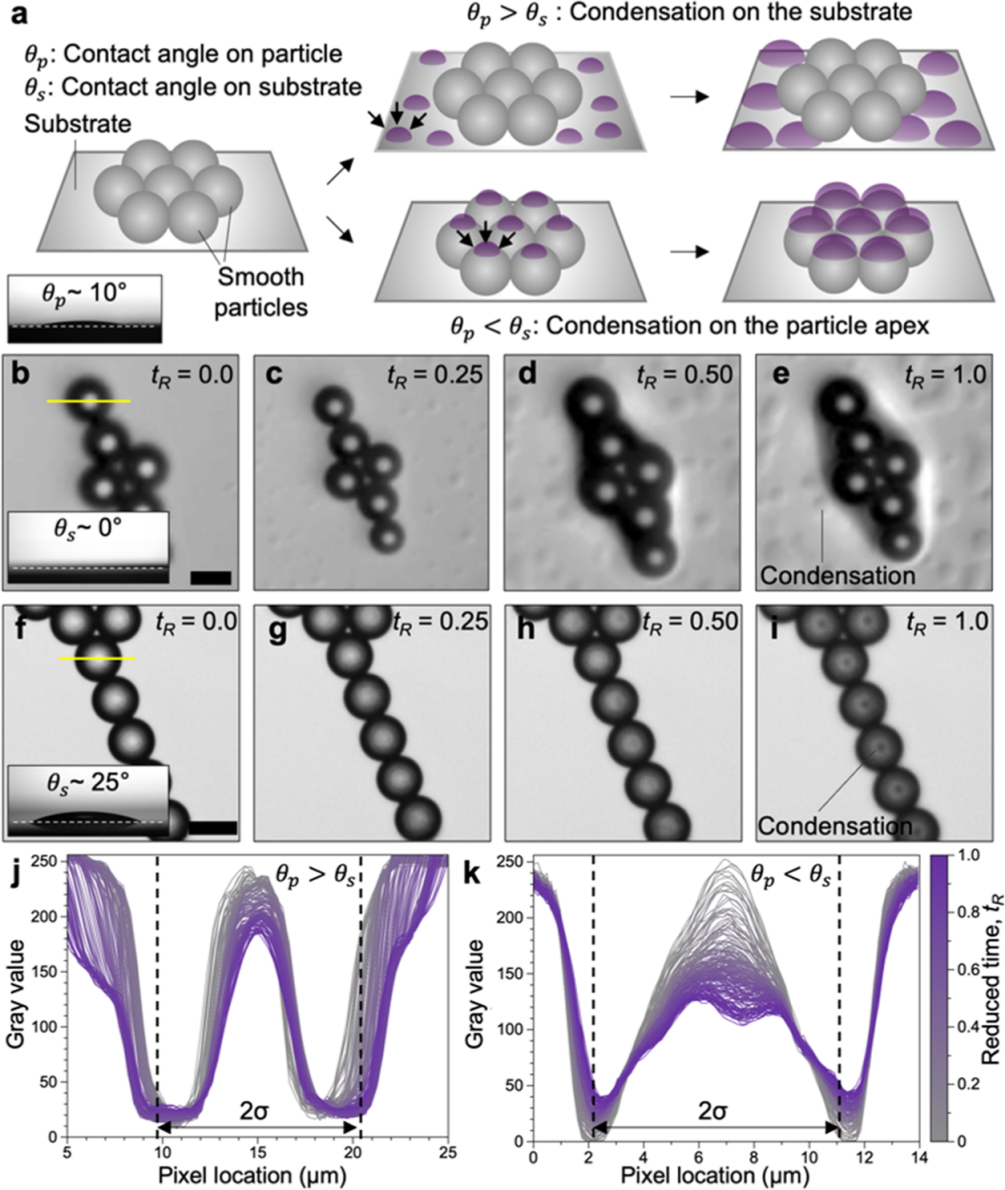
(a) Schematic representing the dependence of the acetone–water vapor condensation behavior on the relative wettability of the particle and the substrate. The condensed droplets are always formed on the more wettable surface, which can be either the substrate containing the PS particles or the PS particle surface. The inset is an image of the liquid droplet of the acetone–vapor mixture on the PS particle-coated silicon wafer, showing the particle contact angle of θ_p_ ∼ 10°. (b–e) Optical microscope images showing the condensation of acetone–water vapor on the substrate when θ_p_ > θ_s_, i.e., when the substrate is more wettable than the PS particles. (f–i) The condensation of acetone–water vapors occurs on the apex of the smooth PS particles when θ*_p_* < θ_s_, i.e., the particle is more wettable than the substrate, which leads to selective polymer corrugation and rough patch formation. Scale bars in (b) and (f) are 5 μm. The insets in (b) and (f), respectively, show the contact angle of the acetone–water mixture on the glass substrate used for respective experiments. (j–k) The changes in the gray values with time across the PS particles as shown by the yellow line in (b) and (f), respectively. No significant changes in the gray values at the particle apex for θ_p_ > θ_s_ combined with droplet formation on the substrate indicate the absence of condensation on the particles, whereas the gray value across a particle decreases and shows a splitting behavior for θ_p_ < θ_s_ indicative of the formation of a droplet at the surface of the PS particle. The reduced time (*t*_R_) is used instead of real time, as the kinetics of the condensation under the microscope and in the setup shown in Scheme 1 are not identical. The acetone–water composition for all of the experiments shown in (b–k) is ϕ_L_ = 0.36.

The formation of rough patches on PS particles is governed by the wettability characteristics of the particles and the substrate (Figure 2a).^49−51^ We demonstrate such dependence by performing experiments on transparent glass substrates of dissimilar wettability while observing the vapor condensation process using an optical microscope. We use two glass substrates with acetone–water mixture contact angles (θ_s_) of ∼0 and ∼25° (insets in Figure 2b,f). Our experiments used commercially available PS microbeads (Spherotech, Inc.) of σ = 5.0 μm as model particles to enable *in situ* observation of the condensation process, which was not feasible for σ = 1.1 μm PS particles due to limitations in optical resolution. The contact angle of the acetone–water mixture on the synthesized and commercial microbeads (θ_p_) was ∼10° (inset, Figure 2a). We find that for ϕ_L_ = 0.36 when θ_p_ > θ_s_, the vapors condense onto the glass substrate instead of on the particle (Figure 2b–e). Such condensation behavior drives the deformation in the shape of the particles instead of patch formation (Figure S9). However, for θ_p_ < θ_s_, acetone–water vapor exposure results in the condensation on the apex of the particles, leading to discrete patch formation (Figure 2f–i). The condensation of the acetone–water vapor on the apex of PS particles is further confirmed by monitoring the change in gray values of pixels along a straight line across the particles for θ_p_ > θ_s_ and θ_p_ < θ_s_. The gray value profile across a particle remains nearly unchanged with increasing time (*t*_R_) for θ_p_ > θ_s_ indicating no significant condensation of the vapors on the particles. However, the gray value across the particle decreases and the maximum shows a split for θ_p_ < θ_s_. The formation of the droplet on the apex of the particle attenuates the transmitted light intensity, leading to a decrease in the gray value and hence confirming droplet condensation at the apex of the particle for θ_p_ < θ_s_. Note that the data reported in Figure 2b–k is in reduced time, *t*_R_, which is the ratio of the initial and final observation time. Such a reduced unit of time is necessary, as the experiments were performed within an optically clear chamber to facilitate the microscopic observation of droplet formation, and the time of condensation in this setup is not identical to our standard experimental setup (Scheme 1 and Figure S2). Regardless, the qualitative information on the location of condensation of solvent vapors remains valid and demonstrates the role of substrate wettability in the selective nucleation process.

The observation of the selective condensation of the droplets on the apex of the particles with θ_p_ < θ_s_ is further corroborated with the existing theory for nucleation onto substrates of known wettabilities. The free energy barrier (Δ*G*) to the condensation and formation of nucleus of acetone–water vapors onto a surface can be determined using Volmer’s classical nucleation theory as^52^
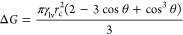
1where θ is the contact angle of the acetone–water mixture on the surface, γ_lv_ is the liquid–vapor surface energy, and *r*_c_ is the critical radius given by Kelvin’s equation (see the Supporting Information (SI) for calculations). The corresponding nucleation rate (*J*) is given by^52^
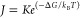
2where *K* is the kinetic constant, *k*_B_ is the Boltzmann constant, and *T* is the temperature. Although Volmer’s theory is primarily intended for flat surfaces, it can still be applied to our case of droplet condensation onto PS particles, as the size of the particles (σ = 1.1 μm) is orders of magnitude larger than *r*_*c*_ (a few nanometers). As can be observed from eqs 1 and 2, the free energy barrier to the nucleus formation as well as the nucleation rate is dependent on the interfacial wettability θ. In our case with θ_p_ < θ_s_ as shown in Figure 2f–i, the free energy barrier (eq 1) for condensing vapors of acetone–water mixture onto the particle apex is ∼40 times smaller than for nucleation onto the substrate under identical thermodynamic conditions. Correspondingly, the rate of nucleation (eq 2) of the acetone-water vapor mixture on the apex of the microparticle is ∼10^16^ times the nucleation rate on the silicon wafer substrate (Figure 2a). It is the higher wettability of the PS particles over the silicon wafer/glass substrates that enables the selective condensation of the acetone–water vapor mixture on the apex of the particles leading to the formation of rough patches.

### Controlling Patch Size and Particle Shape

**Figure 3:**
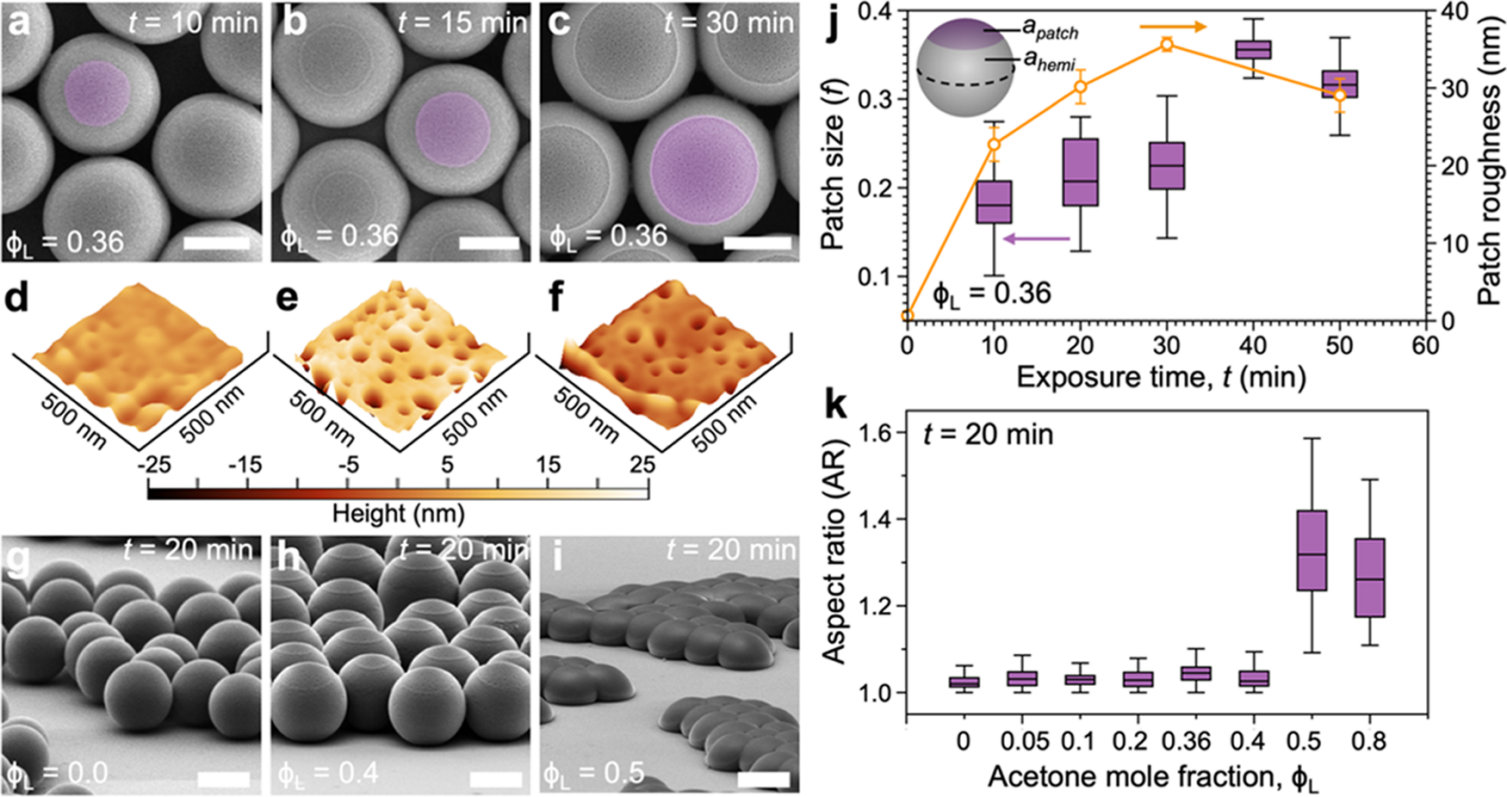
(a–c) SEM images showing an increase in the patch size with increasing time of exposure to the vapors of the acetone–water mixture. Here, the molar composition of the acetone-water mixture was fixed at ϕ_L_ = 0.36. (d–f) AFM images show a change in patch roughness with exposure time corresponding to images (a-c). (g-i) SEM micrographs highlighting the change in particle shape when exposed to solvent with increasing fraction of acetone for *t* = 20 min. The particles show significant deviation from a sphere shape to a “Hawaiian roll” shape at ϕ_L_ = 0.5. The scale bars in (a–c) and (g–i) are 1 μm. (j) Increase in the fractional patch area (left ordinate) with increasing exposure time *t* at constant ϕ_L_ = 0.36. The nonmonotonic change in the patch roughness with *t* is shown by the orange circles, as determined by using AFM (right ordinate). (k) Change in the aspect ratio of particles when exposed for 20 min to vapors containing increasing amounts of acetone. The values of *f* and AR are obtained by analyzing SEM images, where the vertical bars represent the range and the horizontal line is the median of the obtained data set.

The condensation of acetone–water droplets on the apex of PS microparticles is followed by a local swelling process in which the polymer at the apex of the particle partially disentangles and attains a gel state. After removing the PS particles from the experimental chamber, the swollen polymer shrinks, leading to the formation of the observed rough patches. To control the patch characteristics, we investigate the changes occurring in the patch and particle with increasing *t* and varying acetone–water composition. We find that the patch size increases with increasing *t*, from 5 to 50 min at fixed ϕ_L_ = 0.36 (Figure 3a–c,j). We quantify the change in patch characteristics using fractional patch area *f* = (1/2)(*a*_patch_/*a*_hemi_), where *a*_patch_ and *a*_hemi_ are the 2D projection areas of the patch and the particle hemisphere as determined using SEM. The factor 1/2 is introduced to correct for the nonpatchy hemisphere of the particle not visualizable in the SEM. We observe a near-linear increase in the patch size upon increasing exposure time (Figure 3j). The increase in the patch size can be attributed to the known linear increase in the size of droplet condensate with time formed on the surface of the PS particle.^53^ We observe a weak dependence of the roughness of the patch on *t* (Figure 3d–f). Here the patch roughness is defined as the maximum roughness measured by the AFM height profile, which includes the valley-like features formed on the patches (Figure 3d–f). The increase in patch roughness with *t* (<50 min) can be attributed to the comparable time scales of droplet condensation (and growth) and the disentanglement of polymer chains at ϕ_L_ = 0.36. The roughness shows a decrease at *t* = 50 min, which is due to the diffusion of the solvent within the polymer network of the particle leading to its “melting” and "Hawaiian roll-type" structure formation (also observable in SEM Figure 3i). The characteristic time for particle shape deformation decreases with increasing ϕ_L_.

The smallest size of the particle where the droplets would condense, corrugate the polymer, and drive patch formation will be dependent on the nucleation and growth rates of the droplet. These rates and corresponding patch formation will be governed by the composition of the liquid, surface tension, temperature, and saturation ratio as well as by the rate of polymer disentanglement. Based on the observations of the change in patch size and roughness (Figure 3), we can approximate the lower limit of the particle size to be of the order of ∼200 nm (radius), below which the condensed droplet with engulf the particle before it could corrugate the particle apex.

The shape of the particle is strongly dependent on ϕ_L_ and increasing the acetone fraction in the liquid phase leads to particle deformation or “melting” due to solvent diffusion within the particle (Figure 3g–i). To demonstrate the effect of the solvent composition, we monitor the aspect ratio of the particles at *t* = 20 min and vary ϕ_L_ in the range of 0.05–0.8. Upon increasing ϕ_L_ from 0.05 to 0.40 we do not observe any significant change in the aspect ratio of the particles. However, for ϕ_L_ > 0.40, the particles are deformed along all three axes into a “Hawaiian roll” shape (Figure 3i). The degree of deformation is quantified by measuring the aspect ratio (AR) of the particles using SEM images. Here, the aspect ratio is defined as AR = *L*_∥_/*L*_⊥_, where *L*_∥_ and *L*_⊥_, respectively, are the characteristic sizes of the particle parallel and perpendicular to the substrate. The AR increases with increasing fraction of the acetone in the acetone/water mixture (Figure 3k). Above a critical value of ϕ_L_ > 0.40 (for *t* = 20 min), the acetone in the droplets condensed at the apex of the particles is concentrated enough to induce significant particle swelling. While complete dissolution does not yet occur, the solvent penetrates the PS particle more effectively, causing the expansion of the polymeric network, and the spherical shape is lost (Figure 3i). We further investigate the impact of solvent composition on the particles by measuring the height profile and roughness of the patch as a function of ϕ_L_ at *t* = 20 min (Figures S7–S8). No discernible difference is observed between the height profiles or patch roughness on the particles as the acetone molar fraction is varied, likely due to lack of significant differences in the polymer chain disentanglement rates in the tested solvent quality i.e., φ_L_. Further studies focusing on the dynamics of the polymer chains at varying φ_L_ are necessary to fully understand the origin of such invariability in patch roughness.

The swelling process interconnects the neighboring particles, forming a single unit (Figures S10–S11). The linking is attributed to the interwinding of the molecular network of neighboring particles while the polymer is in its swollen state, which does not retract upon halting the swelling process or ceasing the fusion process by opening Chambers 1 and 2 (Scheme 1). Note that using pure acetone completely melts the PS particles, even at *t* < 3 min and no patch formation was feasible. In this work, acetone was mixed with water to reduce the acetone concentration in the liquid and the vapor phases, allowing for reduced solubility of the polymer and leading to patch formation.

### Assembly of Patchy Particles

**Figure 4:**
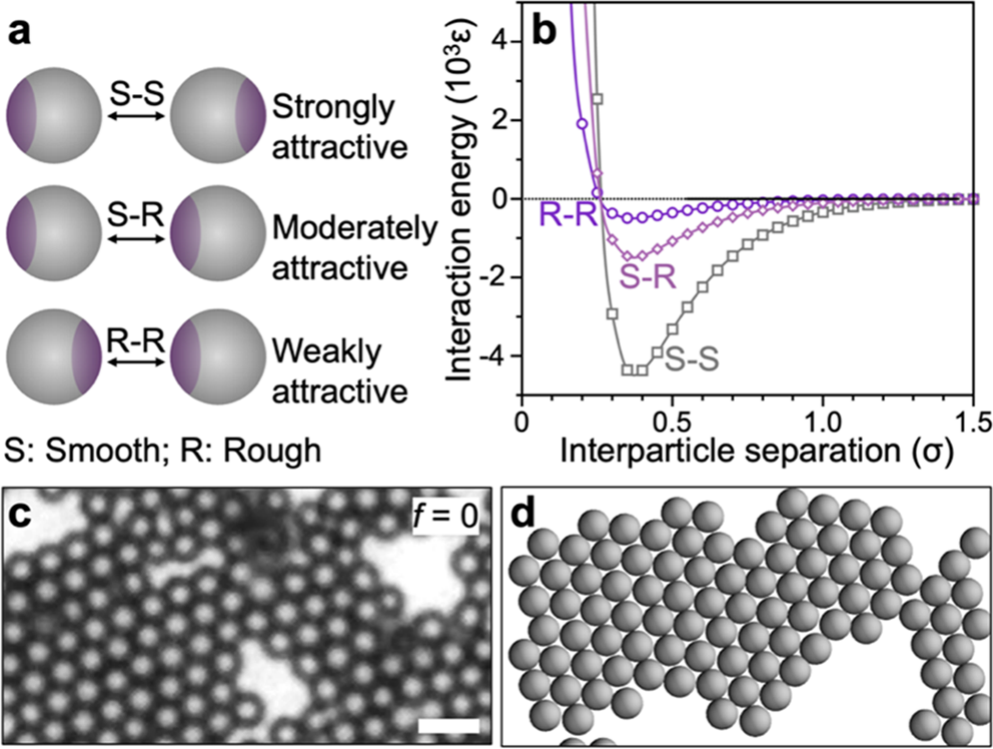
(a) Schematic representation of the qualitative nature of interactions between the smooth–smooth (S–S), smooth–rough (S–R), and rough–rough (R–R) sections of a pair of patchy particles. (b) Interaction energy between a pair of patchy particles with S–S, S–R, and R–R orientations shown in (a). The interaction energy is estimated by summation of the pairwise interactions between the individual points forming the patchy microparticles. (c) Micrograph showing the depletion-attraction-induced crystallization of smooth PS microparticles. Here sodium alginate is used as the depletant. (d) Snapshot of coarse-grained MD simulations showing the formation of 2D *hcp* crystals as the equilibrium assembled state of the smooth particles observed in experiments. Scale bar in (c) is 5 μm.

The formation of rough patches on the surface of PS particles impacts their equilibrium self-assembled state. Previously studies have demonstrated an increase in the roughness of interacting surfaces reduces the effective depletion attraction.^33,35−37,41,54^ In our case, the roughness is localized to a small region on the surface of the particle and, thus, influences the structure of the assembled states. Depending upon the relative orientation of the two interacting patchy colloids, the following three pair interactions exist between the two patchy colloids (Figure 4a): (1) smooth–smooth (S–S), (2) smooth–rough (S–R), and (3) rough–rough (R–R). The depletion attraction depth for these interactions follows the order SS > S–R > R–R. We test this hypothesis using the mathematical modeling approach (discussed below) and obtain the pair interaction energy as shown in Figure 4b. Note that since the roughness of the patch is significantly smaller than the size of the particles, the range of interactions in all three scenarios is similar.

**Figure 5:**
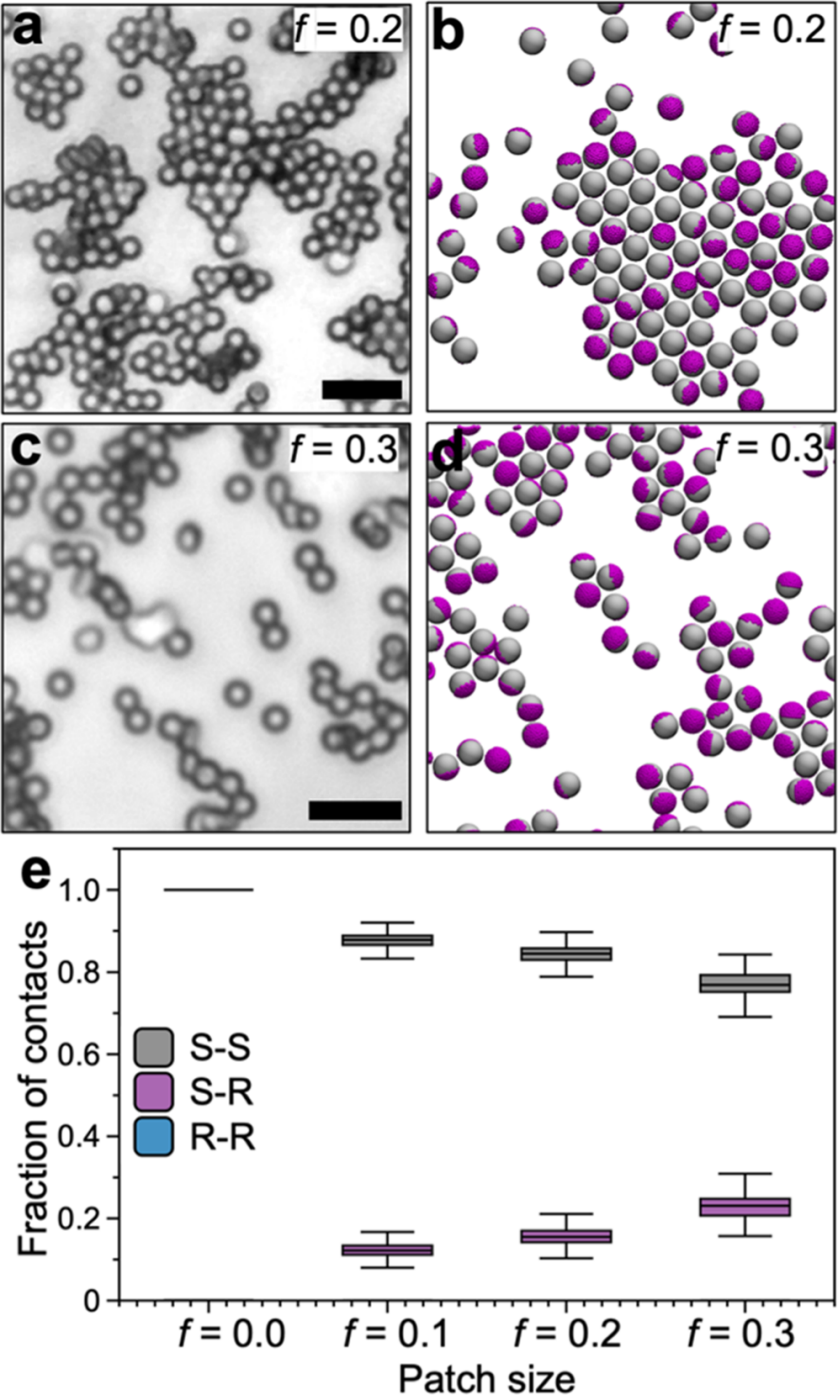
(a, c) Optical microscope images showing the depletion attraction-driven assemblies formed by microparticles with increasing patch size. (b, d) Snapshots of the equilibrium structures obtained in the MD simulations for increasing *f*. Here, the purple regions on the microparticles represent the rough patch. (e) Change in the number of S–S, S–R, and R–R contact points within the equilibrium structures formed by the microparticles with increasing patch size. The number of R–R contacts remains zero in all tested patch sizes. The large number of S–S contact points in nearly all *f* indicate the dominant role of depletion attraction between smooth sections of the neighboring particles in the assembly process. The scale bars in (a) and (c) are 5 μm. In (e), the vertical bars represent the range, and the horizontal line is the median of obtained data set.

We investigate the self-assembly of the microparticles in water using sodium alginate (molar mass ∼222 kDa) as a model depletant. In a typical experiment, the colloidal particles were suspended in an aqueous solution containing 0.1 wt % sodium alginate. The dispersion was inserted into a rectangular glass capillary with a depth of 100 μm, whose ends were sealed onto a microscope slide using UV-curable glue. The capillary was left undisturbed for 24 h for the assembly process to proceed. The near-equilibrium assembled structures were observed using an optical microscope in bright field mode. In our experiments, we find that isotropically smooth PS particles spontaneously organize into 2D hexagonal closed-packed (*hcp*) structures (Figure 4c). The assembled structures were restricted to the bottom of the assembly chamber due to gravity. Upon introduction of the rough patches, the particles self-assemble into partially ordered fractal-shaped clusters (Figure 5a,c). The assembled state of the particles is dependent on the overall patch size, where the degree of ordering decreases with increasing patch size. The experimental conditions for assembly, i.e., depletant concentration, particle volume fraction, and temperature, are identical for the experiments shown in Figure 4c. Therefore, the change in assembled state of the particles observed in Figure 5a,c can be attributed solely to the presence of a rough patch on the surface of the PS particles.

To better understand the role of rough patches in dictating the assembly process of the colloidal particles, we performed coarse-grained molecular dynamics (MD) simulations. The simulations were performed in a canonical ensemble of 100 particles placed within a 3D box of size 35σ × 35σ × 4σ, where σ is the particle radius. This simulation box size is chosen to capture the necessary 2D characteristics of the assembly process and minimize the complexity which arises in the 3D. In the simulations, each particle consisted of 252 evenly spaced points covering the surface of a central spherical core as previously reported.^29,55^ The points were defined to be either type S or type R representing smooth or rough sections of the particle surface, respectively. The patch on the particle was constructed by assigning a given number of neighboring points (*n*_R_) as type R such that *f* = *n*_R_/252. The pair interactions among the S–S, S–R, and R–R points on neighboring particles are modeled by the Wang–Frenkel potential (see the SI),^56^ with potential energy well depths of ε, 0.3ε, and 0.1ε, respectively. The simulations are performed with a periodic boundary condition in *x* and *y* directions using the LAMMPS package^57^ in reduced units of size, time, and temperature. The simulations were run for 2 × 10^6^ steps and the assembly process was monitored using VMD software.^58^ Further details on the simulations are provided in the SI. In the absence of a rough patch, the microparticles self-assemble into highly ordered crystals with 2D *hcp* as shown in Figure 4d. Similar to the experiments, in the absence of the rough patch, the simulations show the formation of 2D crystals confined to the bottom of the simulation cell, validating the simulation approach. We first calculate the interaction energy between a pair of patchy particles with *f* = 0.3 in three fixed configurations shown in Figure 4a, and the corresponding interaction energies are shown in Figure 4b. As hypothesized above, the depth of the interaction energy well between a pair of patchy particles follows the order S–S > S–R > R–R. This asymmetry in the surface interactions between particles drives the change in the self-assembled state of the patchy colloidal particles.

Experiments show the introduction of the rough patch leads to the disruption of the 2D *hcp* structure and increasing the patch size drives the formation of 3D fractal clusters. The simulations show fewer particles assembled upon increasing *f* (Figure 5b,d), which is in agreement with the experimental observations (Figure 5a,c). We perform semiquantitative comparison between the cluster size distribution obtained in the experiments and simulation, which show a reasonable agreement (Figure S12). Note that complete quantitative comparison between experiments and simulation is not feasible for two reasons: (1) Only a finite number of particles (here 100) that can be simulated, which could lead to a smaller average cluster size than experiments; and (2) large variability in the local number density of the microparticles within the experimental chamber, driving the large standard deviations in average cluster sizes.

We identify the change in the fraction of S–S, S–R, and R–R contacts in our simulations for a given *f* by monitoring the distances between R, and S points of neighboring particles as detailed in the SI. At equilibrium, the fractions of S–S, S–R or R–R are dependent on the patch size *f* as shown in Figure 5e. We find that the fraction of S–R contacts increases with *f* but that R–R remains zero. Despite the increase in the S–R contacts, the fraction of S–S contacts remains dominant at *f* ≤ 0.3. Such predominance of the S–S contacts is due to the higher attraction strength between the smooth sections of the particles. The absence of R–R contacts for nearly all tested *f* indicates that the R–R contacts are sacrificed for the formation of more preferential S–S and S–R contacts. Upon increasing *f*, the disappearance of highly ordered structures restricts the quantitative comparison between assemblies observed in experiments and MD simulations. Regardless, two key conclusions can be made from the MD simulations: (1) Introducing discrete patches with reduced interaction energy (here roughness) allows altering the self-assembled state of the particles; and (2) The S–S and S–R interactions play the governing role in directing the self-assembly of the patchy particles into discrete clusters. Further experiments and MD simulations will allow identifying the roles degree of roughness, shape, and size of patch on the assembly process.

### Conclusions

The study provides a simple, robust, and precise method to fabricate polymeric microspheres with a well-defined rough patch for the first time. The rough patch is introduced by selectively nucleating and condensing vapors of an acetone–water mixture on the apex of a smooth PS particle immobilized onto a substrate. The vapor condensation on the particle and patch formation occur only when the particles are more wettable than the substrate containing the particles. The selective condensation of the acetone–water mixture, i.e., the good solvent at the apex of the particle, leads to the polymer corrugation and formation of a rough patch on a smooth PS particle. Patch size and particle shape can be controlled through the VLE relationship of the solvent by altering the acetone concentration as well as the exposure time of the particles. In the presence of a depletant, the patchy PS particles self-assemble into discrete clusters, which differs from the spontaneous crystallization of the smooth PS particles. The MD simulations reveal that the assembly is driven by the preferential smooth–smooth surface pair interactions, and the morphology of the resulting clusters formed is governed by the presence of rough patches. Overall, the study establishes a method of synthesizing spherical colloids with discrete rough patches of desired size, which has been a long-standing challenge. We anticipate that the present approach can be extended to any polymeric particle-good solvent pair, given that the good solvent has a large vapor pressure. The fabrication method could be potentially scaled up through the implementation of a continuous conveyor mechanism within a controlled vapor–liquid equilibrium chamber, thus providing new opportunities for the large-scale production of polymeric microspheres with tailored patch roughness. Furthermore, the availability of the model colloidal particles with rough patches of tunable size opens new opportunities to investigate and understand assembly processes at the molecular and nanoscale.

## Methods

### PS Particle Synthesis

Styrene (Sigma-Aldrich, stabilized for synthesis), 2,2′-azobis(2-methylpropionitrile) (AIBN) (Sigma-Aldrich), and polyvinylpyrrolidone (PVP) (VWR, high-purity grade, MW∼ 40 kDa) were used as supplied. PS spheres were prepared via dispersion polymerization using AIBN as the initiator.^45−47^ Under magnetic stirring, 44 mL of ethanol and 0.9 g of PVP were added to a 250 mL round-bottom flask and heated to 70 °C. Styrene (28 g) was then added to the flask, and the system was deoxygenated with nitrogen gas. After 15 min, 0.3 g of AIBN dissolved in an additional 44 mL of ethanol was added, and the mixture was once again purged with nitrogen gas. Stirring was set to 180 rpm, and the solution was allowed to polymerize for 24 h. The mixture was cooled to room temperature, and the obtained microspheres were removed through repeated washing/centrifugation/redispersing in ethanol and water. The microspheres were stored in an aqueous solution until use.

### Optical Microscopy and SEM

The synthesized smooth microparticles were characterized for their size, shape, and polydispersity by using bright field optical microscopy performed on an upright Leica DM6000 microscope. The particle size distribution was determined by image analysis of ∼1000 particles using the ImageJ software package. Scanning electron microscope (SEM) imaging was performed on an FEI Quanta 3D FEG FIB/SEM at the Shared Instrumentation Facility (SIF) housed at Louisiana State University. All samples were coated with a thin layer of platinum by using a plasma sputter coater prior to imaging unless otherwise stated.

### Atomic Force Microscopy (AFM)

The nanoscale surface roughness of both smooth and patchy PS particles was assessed by using AFM (Bruker Dimension FastScan). The AFM is equipped with a silicon cantilever tip on a nitride lever (SCANASYST-AIR, *f* = 70 kHz, *k* = 0.4 N m^–1^) and used in tapping mode. Subsequently, all collected data was processed using Gwyddion SPM analysis software package,^59^ following a consistent protocol. The data processing steps included the removal of a second-degree polynomial background from the images to account for the curvature of the microparticles, thus enabling the accurate measurement of surface roughness. Additionally, a Gaussian blur and color scale were applied to enhance clarity in the visual representation (Figure S13). Note that the roughness values reported here are the maximum roughness values that include and represent the troughs formed on the patch as shown in AFM and SEM measurements.

### ATR-FTIR

To identify the differences in the chemistry of the smooth and patchy particles, the vibrational spectra were obtained using a monolithic diamond crystal ATR accessory on a Bruker α FTIR instrument. After the instrument was blanked with air, measurements were taken by collecting 32 scans per spectrum at a 4 cm^–1^ resolution. In our experiments, we find that despite the distinct physical properties of the patch, the vibration spectra of the pristine and patchy PS particles remain nearly identical (Figure S4).

### NMR

^1^H NMR spectra were recorded on a Bruker Avance III 400 MHz spectrometer at 298 K. The samples were prepared at a concentration of 10 mg/mL in deuterated chloroform (CDCl_3_). Chemical shifts (δ) are given in parts per million (ppm) and referenced to the deuterated solvent signal.

### Size Exclusion Chromatography

The SEC was performed on a TOSOH HLC-8320 GPC instrument equipped with a TSKgel superH5000 column (3 μm particle and 20 nm pore size). The number-averaged molecular weight (termed molas mass) was determined relative to linear polystyrene standards.

### Depletion-Driven Assembly Experiments

A suspension was prepared containing either smooth or patchy PS microparticles, 0.1 wt % sodium alginate (Ward’s Science) to introduce depletion interactions. The suspension was contained in flat borosilicate capillaries (VitroTubes). For all experiments, the concentration of PS particles in the suspension was adjusted to cover approximately 20% of the available surface at the bottom of the capillary. Each end of the filled capillary was sealed with UV-sensitive glue and cured onto a microscope slide with UV light (Uvitron).

## Supporting Information Available

Microparticle size distribution, 3D model of the experimental setup, VLE for acetone–water mixture, particle swelling in acetone–water mixture, ATR-FTIR and size exclusion chromatogram, SEM images of the deformed particles upon prolonged exposure, or preferential wetting of the substrate, SEM and optical micrographs showing interlinking of particles, additional AFM measurements, height profiles, roughness estimations, calculations of the critical radius, and details of the MD simulations (PDF)
