# Supplementary material for: Selective Vapor Condensation for the Synthesis and Assembly of Spherical Colloids with a Precise Rough Patch
Source: JACS Au. 2024 Mar 4;4(3):1107–17. doi: 10.1021/jacsau.3c00812 (PMC10976603; doi:10.1021/jacsau.3c00812)
Supplement: Supplementary file 1 — au3c00812_si_001.pdf [file au3c00812_si_001.pdf]

Supplementary Information for

Selective vapor condensation for the synthesis and assembly of spherical colloids with a precise rough patch

Kennedy A. Guillot[1,†], Philip J. Brahana[1,†], Ahmed Al Harraq[1,‡], Nduka D. Ogbonna[1], Nicholas S. Lombardo[1], Jimmy Lawrence[1], Yaxin An[1], Michael G. Benton[1], and Bhuvnesh Bharti[1,*]

[1]*Cain Department of Chemical Engineering, Louisiana State University, Baton Rouge, LA, 70803, USA*

[,†]These authors contributed equally to the work.

[‡]Present address: *Center for the Physics of Biological Function, Princeton University, Princeton, NJ 08544, USA*

*Corresponding author: bbharti@lsu.edu

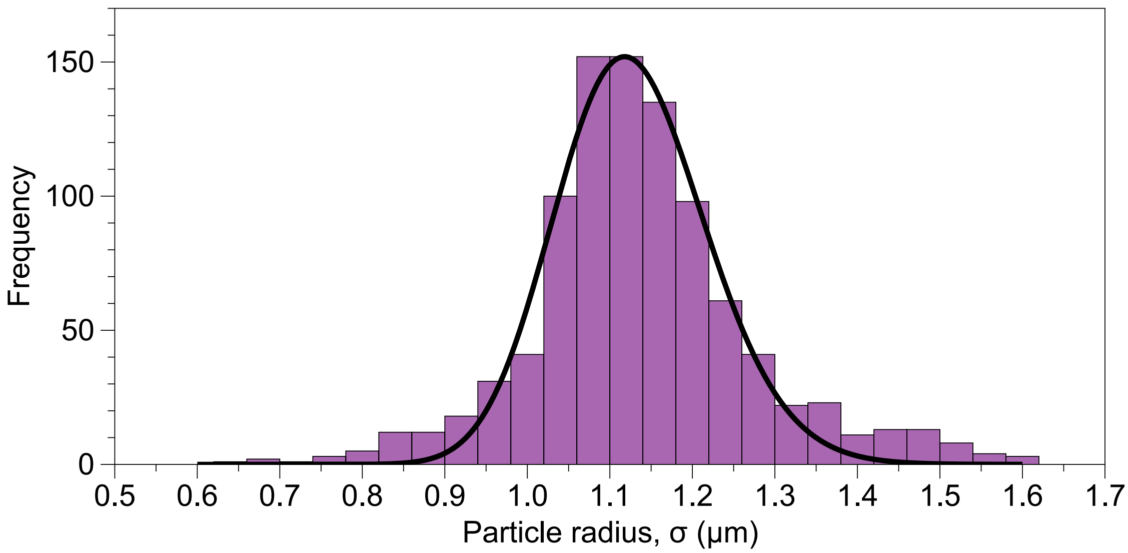

Figure S1. Size distribution for the synthesized PS particles used for experiments. The frequency distribution is obtained analyzing the optical microscope images. The bars represent the measured data, and the line represents the fit to the data using log-normal distribution. The particles show a mean radius of ~1.1 μm.

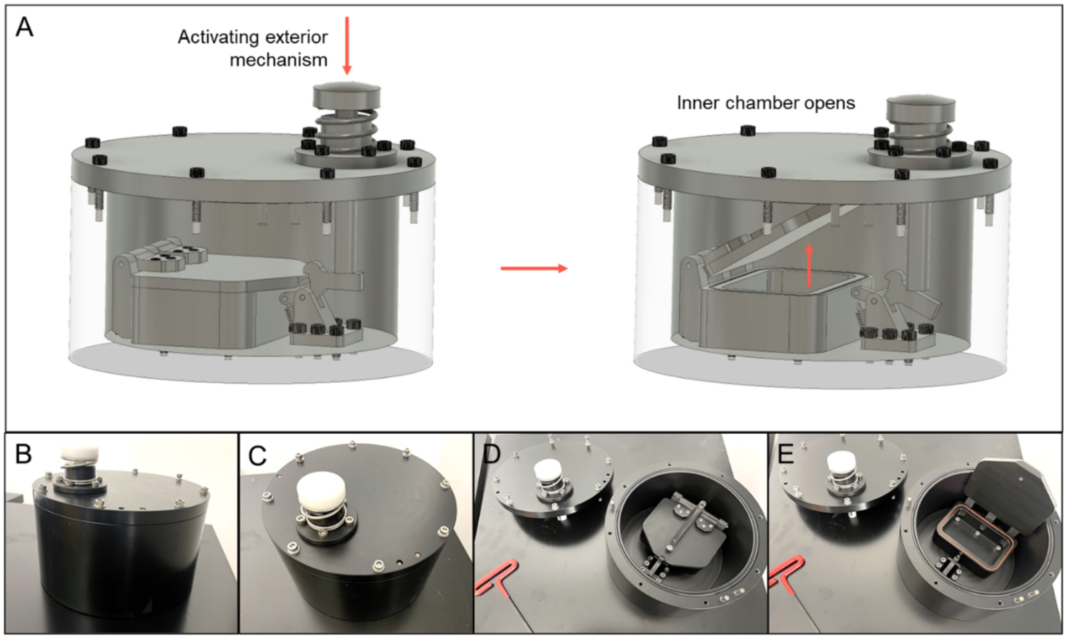

Figure S2. 3-D model and photographs of dual chamber system used in our experiments. (A) Scheme depicting the mechanism allowing the inner Chamber 1 to open while the outer Chamber 2 remains sealed. This allows for the acetone-water mixture to remain in equilibrium while controlling particle exposure. (B-C) Exterior of the outer Chamber 2. (D-E) Interior of the outer chamber with a closed (D) and open inner Chamber 1 (E).

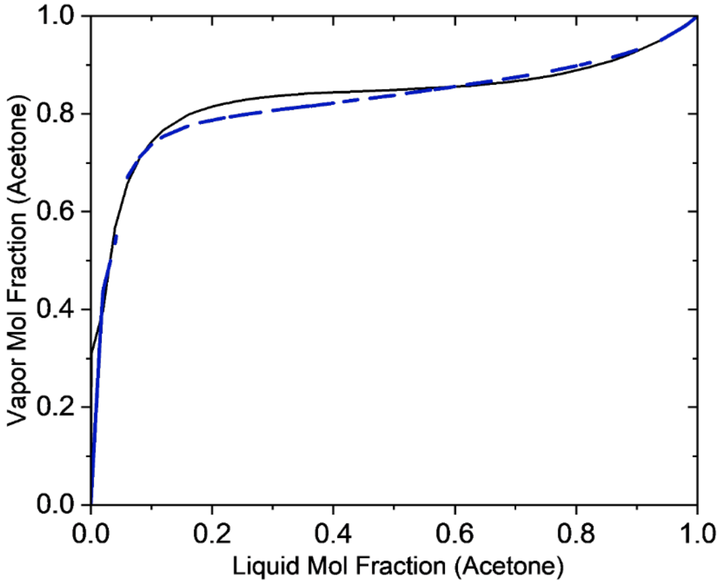

Figure S3. Vapor-liquid equilibrium (VLE) relation for acetone-water mixtures. This relationship was simulated through Aspen+ utilizing the National Institute of Standards and Technology (NIST) databanks available. Graphs were generated both through the NRTL (Non-random two-liquid) (black, solid line) and UNIFAC (UNIQUAC functional-group activity coefficients) (blue, dotted line) property methods. For experimentation, liquid composition of each component was directly measured, and vapor composition was estimated through this relationship.

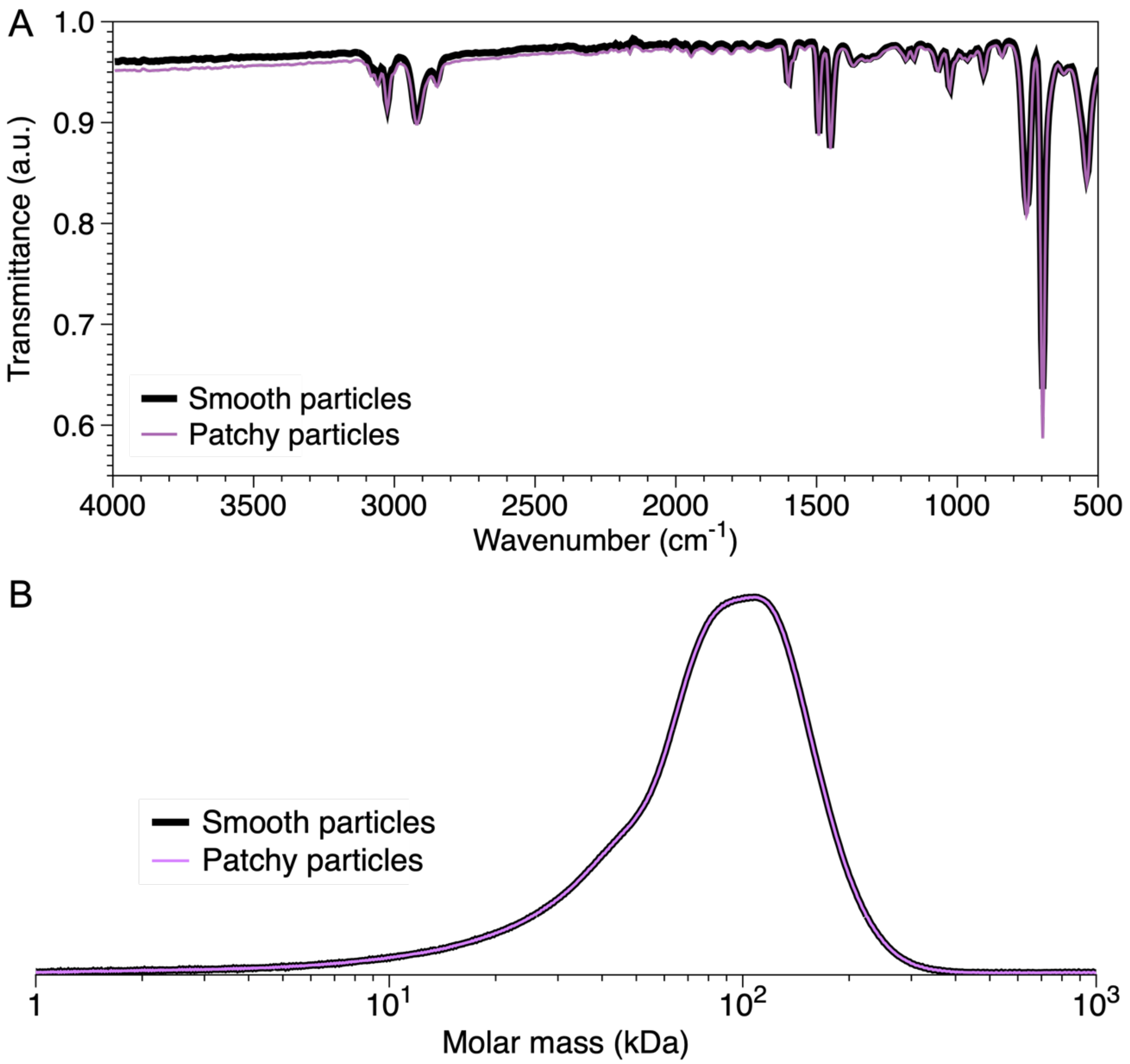

Figure S4. (a) ATR-FTIR of the smooth and rough PS particles. The spectrum shows no significant chemical change in the particles upon introduction of the rough patch. (b) Size exclusion chromatogram (SEC) showing the identical molar mass distribution of the polymer forming the smooth and patchy particles.

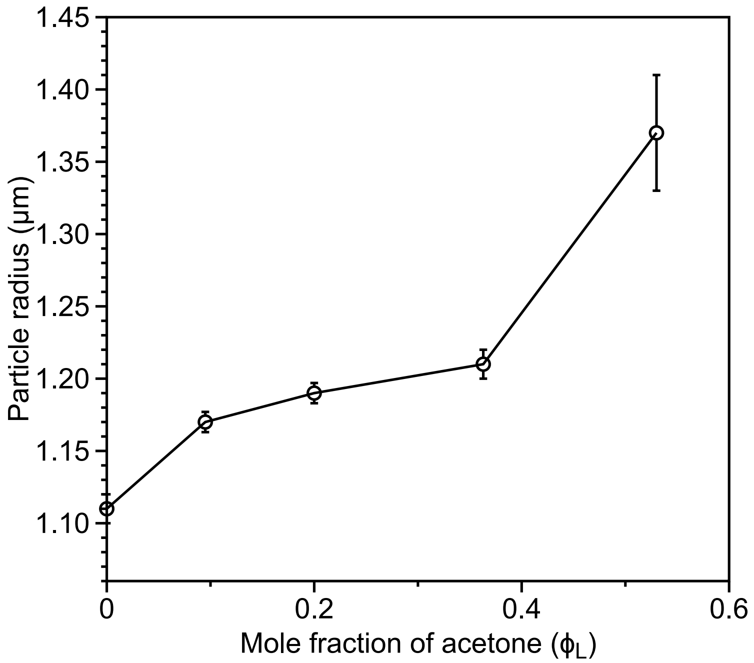

Figure S5. Increase in the radius i.e. swelling of the non-crosslinked PS particles upon changing the fraction of the acetone present in the continuous medium. The change in size was determined using optical microscopy. The circles are the mean of the measured values for a set of at least 1500 particles, and bars represent the standard error. The particles show shape deformation beyond $\phi_L>0.4$, and thus were not used for estimating particle swelling. Note that this swelling was measured by dispersing the PS particles in the liquid acetone-water mixture.

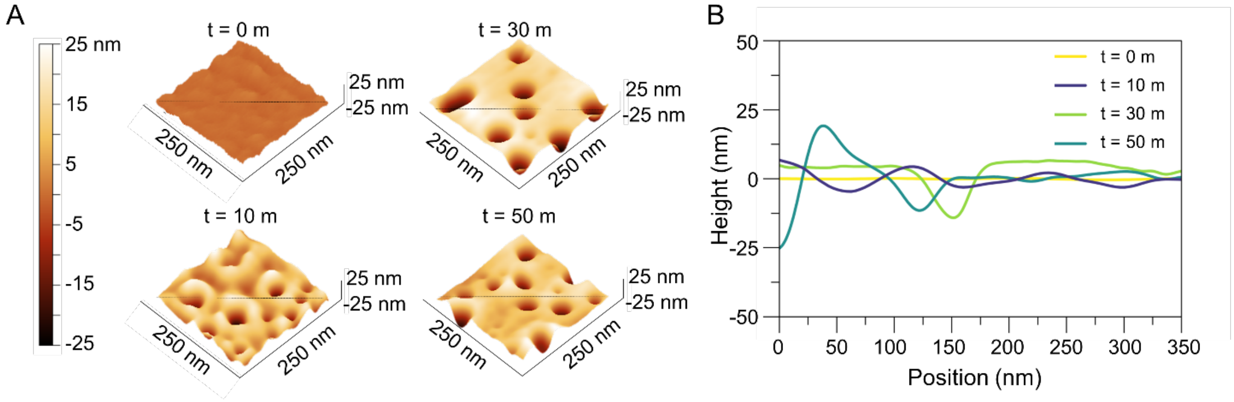

Figure S6. AFM images and height profiles of particles at various exposure times (t = 0, 10, 30, 50 min). (A) Shows the 3D reconstruction (via AFM) of polystyrene particle surfaces following exposure to acetone-water vapors for different durations. (B) Depicts the corresponding surface profiles at each time point. The x-axis represents the position of the cantilever on surface, while the y-axis represents the height traversed by the cantilever.

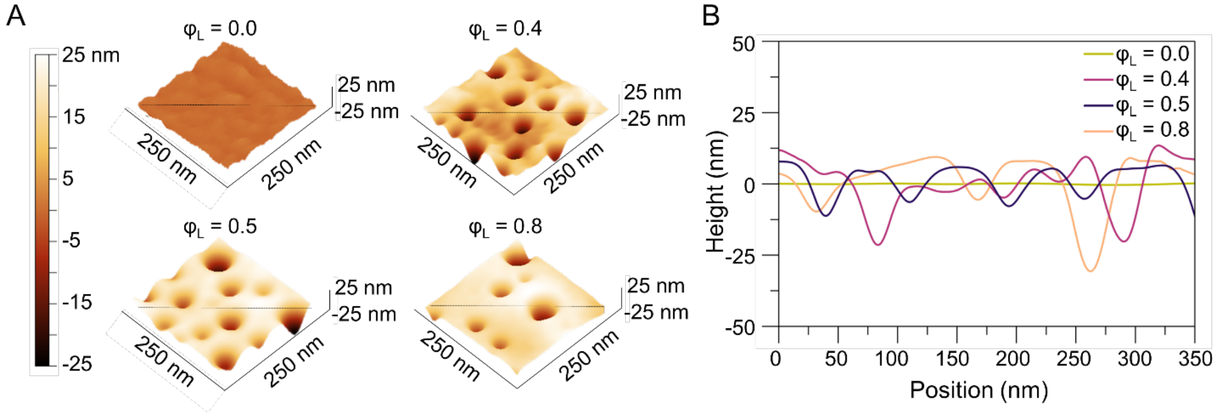

Figure S7. AFM images and height profiles of particles at various acetone molar fractions (φ_L = 0.0, 0.4, 0.6, 0.8). (A) Illustrates the AFM 3D reconstruction of polystyrene particle surfaces subjected to varying acetone-water mixtures. (B) Depicts the corresponding height profiles at each molar fraction. The x-axis indicates the distance covered by the cantilever across the surface, while the y-axis represents the height traversed by the cantilever.

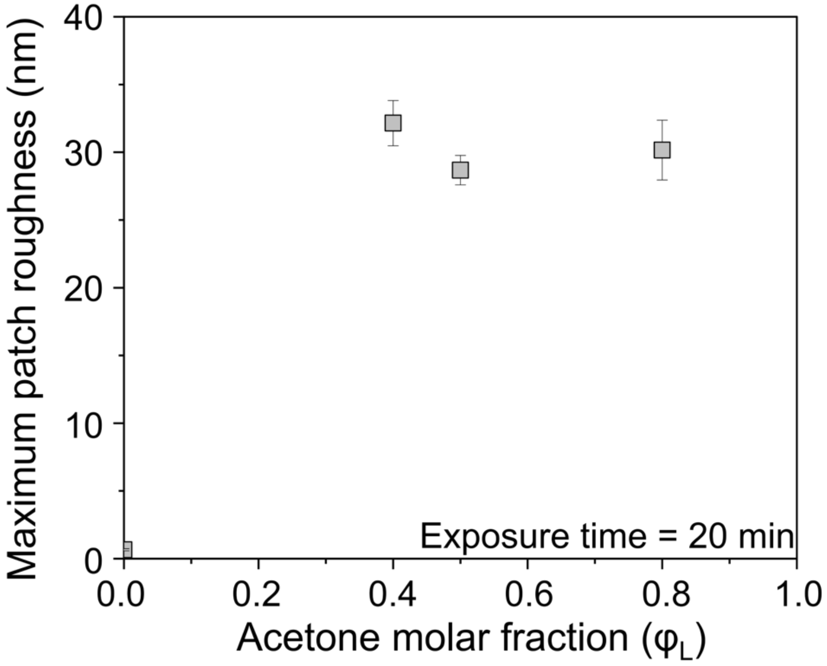

Figure S8. Roughness of patches at various acetone molar fractions (φ_L = 0.0, 0.4, 0.6, 0.8). The symbols depict the maximum roughness of the patches based on over ten measurements. The error bars indicate the standard error. Each group underwent exposure to the corresponding acetone vapor for a duration of 20 minutes.

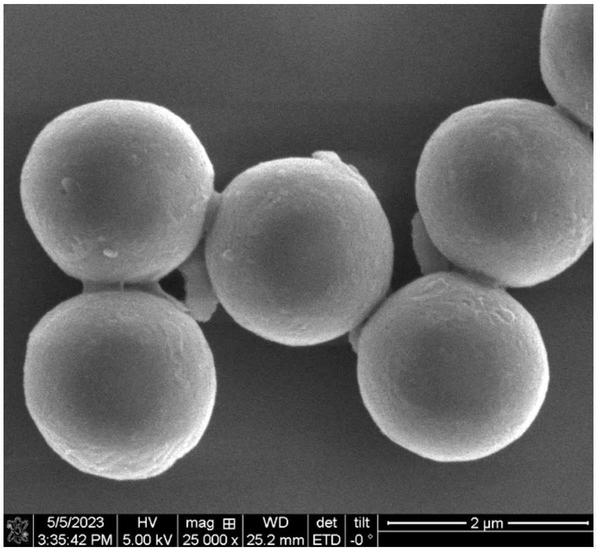

Figure S9. SEM image showing the deformation in the shape of the particles when the substrate was more wettable than the particles, i.e. $\theta_p > \theta_s$.

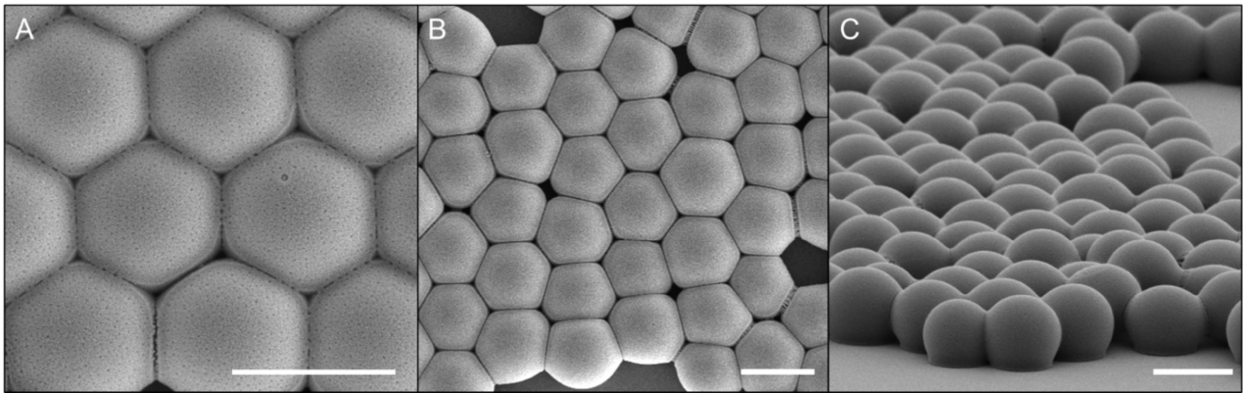

Figure S10. SEM images of PS particles after being exposed to vapors of acetone-water mixture with ϕ_L = 0.36 for *t* = 60 minutes. (A) Top view of particle surface. Rough patch covers entire visible surface. (B) Top view of PS monolayer. Effects of exposure have begun to show signs of shape distortion. (C) Side view of PS monolayer (camera tilted 74°). Aspect ratio begins change by this timepoint. Scale bars represent 2 μm.

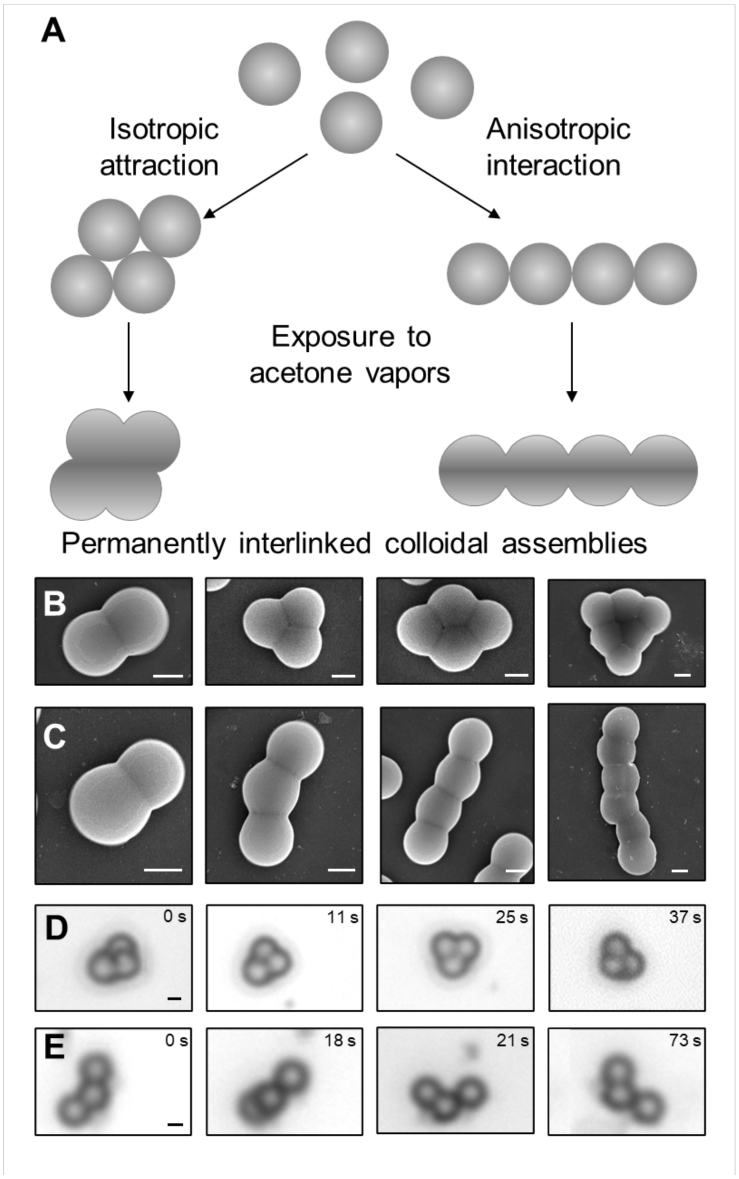

Figure S11. Synthesis of interlinked particle assemblies. (A) Schematic depicting the method for particle interlinking. (B) Particle clusters linked together through isotropic assembly, followed by acetone vapor exposure. (C) Particle clusters linked through anisotropic assembly followed by exposure to acetone vapor. (D, E) Three-particle cluster (D) and chain (E) interlinked and imaged in aqueous solution. Consecutive images display different time points of the same interlinked particle, showing its movement as a single unit. Scale bar represents 1 μm.

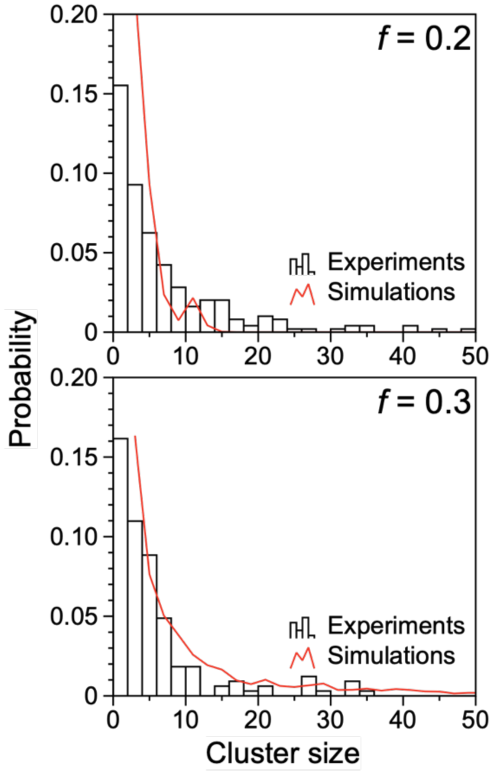

Figure S11. Cluster size distribution obtained for the experiments and simulations for patch sizes *f* = 0.2 and 0.3. Here the cluster size is defined as the number of particles within a cluster.

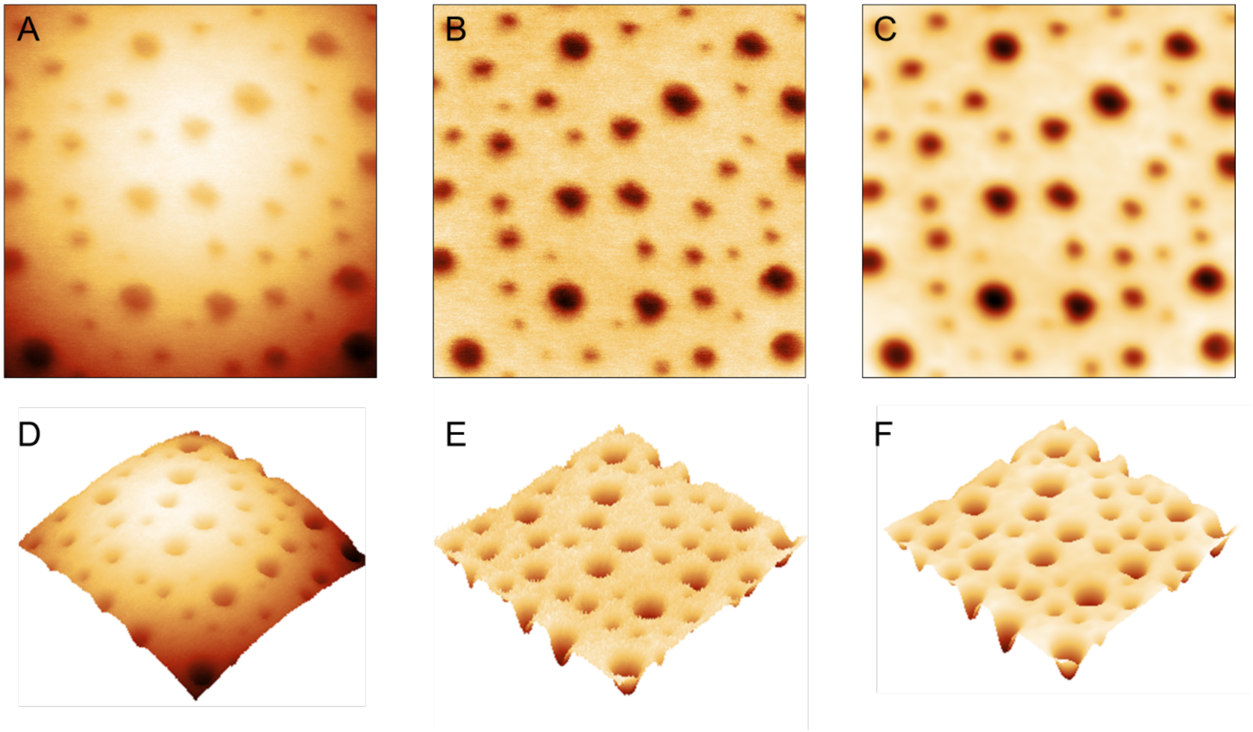

Figure S13. Data processing performed using Gwyddion SPM analysis software. Panels (a-c) display the progression from raw AFM scan images to processed representations. (a) Presents the initial raw image captured by the AFM. (b) Illustrates the image post second degree polynomial background removal, and (c) showcases the image following a 5-pixel Gaussian blur application. Corresponding 3D reconstructions at each processing step are presented in panels (d-f).

Calculation of the Critical Radius (*r_C*)

The critical radius (r_C) refers to the minimum size of a stable droplet, and it is where the free energy of the nucleus formation has a local maximum. To calculate r_C, we use Kelvin's equation

$$r_c = 2\gamma_{lv}V_m/(RT\ln(p/p_\infty))$$

Here γ_{lv} is the liquid-vapor surface energy (or surface tension), p and p_∞ respectively are the vapor pressure over curved surface of radius r_c, and saturation pressure over a flat surface, V_m is molar volume of the acetone-water mixture, R is the universal gas constant, and T (= 293 K) is the temperature. The surface tension and $V_m$of the acetone-water mixture at ϕ_L = 0.36 is ~ 30 mN/m (measured in our lab), and V_m ~ 40 mL/mol (from the Kezic et al.[1]). Assuming a typical saturation ratio of p/p_∞ = 1.7, as the case for previous study on condensation of water onto surfaces[2], the value of r_c is approximately 2 nm."

Molecular dynamics simulations of the self-assembly of nanoparticles

We performed molecular dynamics simulations to understand the mechanism of the self-assembly of the nanoparticles. We simulated 100 particles in a box with a narrow z-direction width (x = 35σ, y = 35σ, z = 4σ) to mimic the effects of gravity. The simulation is periodic in the x and y directions. Each nanoparticle consisted of 252 evenly-spaced points covering the surface of a central spherical core adapted from this website [http://neilsloane.com/icosahedral.codes/], as previously reported. The points were defined to be either type R or type S representing rough or smooth surfaces, respectively. Each particle is treated as a rigid entity in the MD simulations. All the simulations were performed by using the LAMMPS package in reduced units where the characteristic quantities of m, σ, and ε are for mass, distance, and energy, respectively, and the reduced time unit is $(m\sigma^2/\varepsilon)^{1/2}$. The particles have a diameter of 2σ and the patch area if defined as $f = n_R/(n_R+n_S) = n_R/252$, where n_R, and n_S are the number of type R and type S points, respectively. The interactions between R-R, S-R, and S-S beads are modelled by the Wang-Frenkel (wf/cut) potential. The wf/cut potential is a derivative of the Lennard-Jones 12-6 potential developed for systems with a typical short-range potential with attraction as:

$$\phi(r) = 0.2A\alpha\left(\left[\frac{B}{r}\right]^{2\mu} - 1\right)\left(\left[\frac{r_c}{r} - 1\right]\right)^{2\nu}$$

Where $\alpha = 2\nu\left(\frac{r_c}{B}\right)^{2\mu}\left[\frac{1+2\nu}{2\nu\left[\left(\frac{r_c}{B}\right)^{2\mu}-1\right]}\right]^{2\nu+1}$, $\mu = 1$, A is the depth of the potential well, B is the distance at which the potential $\phi(r)$ is zero, r is the distance between beads, and r_c is the cutoff (2σ). Here, for type-R beads A=0.1ε, B=0.5σ, while A=ε, B=0.5σ for type-S beads, and the cross-interaction parameters between R-S are 0.3ε and 0.5σ.

The simulations were performed in canonical ensembles where the temperature was kept at 32.9 $\frac{\varepsilon}{k_B}$ corresponding to a real temperature of 298 K. The temperature was kept constant by using the Nosé–Hoover thermostat. The trajectory was generated by using the velocity-Verlet integration

scheme with a timestep of 0.01 $(m\sigma^2/\varepsilon)^{1/2}$. The simulations were run for 2×10^6 steps. The snapshots of the simulations were created by the VMD software.

The final structure formed by the microparticles was analyzed using types of the contact points between neighboring particles. A contact between a specific pair was defined when the distance between the center of mass of two particles is less than 3σ. Three types of contact: S-S, S-R and R-R are classified by identifying the point types with a minimum distance d_{min} between two contacted nanoparticles. d_{min} is calculated as below:

$$d_{min} = \min\{d_{i,j}\}$$

where $d_{i,j}$ is the Euclidean distance between beads *i* and *j* from each of the particles in the contact pair. The index of *i* and *j* are recorded as *I* and *J* when their distance is minimum. If both beads *I* and *J* are type R, the contact is R-R. If both beads *I* and *J* are type S, the contact is S-S. Otherwise, it's a S-R contact.

REFERENCES

1 B. Kežić and A. Perera, *J. Chem. Phys.* **137**, 134502 (2012).
2 K. K. Varanasi, M. Hsu, N. Bhate, W. Yang, and T. Deng, *Appl. Phys. Lett.* **95**, 094101 (2009).
